# Facilitatory Effect of Extending the Course Duration on Dissemination of Educational Content

**DOI:** 10.1007/s40670-022-01563-4

**Published:** 2022-05-07

**Authors:** Hiromasa Satoh, Fuminobu Tamalu, Narumi Hirosawa, Hajime Hirasawa, Mitsuo Nagane, Ryohei Saito, Shu-Ichi Watanabe, Naofumi Miwa

**Affiliations:** 1 Department of Physiology, Saitama Medical University, 38 Moro-hongo, Moroyama-machi, Iruma-gun, Saitama, 350-0495 Japan; 2 Division of Analytical Science, Biomedical Research Center, Saitama Medical University, Moroyama-machi, Saitama, Japan; 3 Department of Obstetrics and Gynecology, Graduate School of Medicine, Saitama Medical University, Moroyama-machi, Saitama, Japan

**Keywords:** Physiological practice course, Course duration, Self-assessment questionnaire, Likert score

## Abstract

The physiological practice course at Saitama Medical University provides students with the opportunity to learn physiological principles through wet labs and discussions. To develop a more effective method for maximizing learning outcomes, we extended the course’s schedule from one day (1d) to two days (2d) per theme, evaluated self-administered questionnaires between two different years (pre and post-change), and examined whether the increased course length affected learning outcomes. Within the 2018 curriculum year, every theme of the course was completed in a day, including experiments in the wet lab and discussions. In 2019, each theme was assessed for two days. The second-year undergraduate medical students anonymously submitted the self-assessment questionnaire that addressed several aspects, such as understanding of the theme, through a 5-point Likert scale. The average Likert scores varied from 4 to 4.5 point for all questions, and significant differences were not found between the 1d and 2d courses. However, the ratio of students with the highest points increased for one question of the 2d course: 43.6% (1d) to 53.4% (2d) for understanding. Further, the standard deviation (SD) values decreased in the 2d course for every question: 0.29 (1d) to 0.15 (2d) for interest, 0.33 (1d) to 0.19 (2d) for understanding, 0.30 (d) to 0.17 (d) for communication, 0.34 (1d) to 0.19 (2d) for general evaluation. This reduction in the SD values indicated that the educational content was imparted more efficiently to students in the 2d course. Thus, we concluded that extending the course time facilitated dissemination of educational content for every theme.

**Supplementary Information:**

The online version contains supplementary material available at 10.1007/s40670-022-01563-4.

## Introduction

Recently, the introduction of several modern learning methods, including simulations, internet-based resources, and smartphone apps (i.e., dry-based methods), has resulted in better skill set development among medical undergraduate students [1–4]. However, experimental activities (i.e., wet-based methods) have also been proven to induce effective understanding of physiological educational content through their interactive approaches [1, 5, 6]. At Saitama Medical University, undergraduate medical students are required to take a physiological practice training course in their second year. This course provides students with the opportunity to learn a set of physiological responses (e.g., conduction of action potential, skeletal or heart muscle contractions, hormonal regulation for reproduction, and homeostatic ionic regulation by urine), through laboratory experiments and discussions with instructors. Students are also expected to attempt trials and errors to obtain unambiguous experimental data by exploiting the Plan-Do-Study-Act cycle [7]. Accordingly, we consider that this course promotes undergraduate students’ deep learning of human physiology and develops research-oriented mind. In addition, it facilitates a solid understanding of pathophysiology, which is undoubtedly essential knowledge for students who are entering clinical clerkships [8, 9]. To our knowledge, many medical universities in Japan [10] and other countries [11–13] incorporate a physiological practice course in their curricula. Despite the benefits of this course, proper organization is problematic due to time restrictions, limited number of skilled instructors, and insufficient laboratory instruments. Therefore, the current challenge for academic staffs, particularly in physiology departments, is finding effective ways to develop the course (e.g., extending the course time length) in order to maximize students’ learning outcomes [3, 9, 13]. In our physiological practice course, we have already made a few pedagogical adjustments. For instance, we schedule the course so that it follows the class of the same theme with the aim of consolidating the physiological knowledge that has been just learned in the classroom. However, further considerations should be assessed to improve the course. Thus, in this study, we changed the schedule, evaluated the self-administered questionnaires between two different years (pre/post-change), and examined whether the increased course time (from 1 to two days per theme) improved and/or affected students’ learning outcomes, including their interest, understanding, and communication. In this study, we changed one resource allocation (course duration: one day to two days) and assessed program effectiveness. Therefore, our study comprised the characteristics of several program evaluation models, including the quasi-experimental design, Input/Process evaluation of CIPP (Context/Input/Process/Product), and the first level of Kirkpatrick’s evaluation model [14].

## Materials and Methods

### Physiological Practice Class Schedule and Themes

At Saitama Medical University, the physiological practice class is taught annually to second-year undergraduate medical students (aged above 19). In 2018, it comprised seven physiological themes; each theme was completed within a day (approximately eight hours) and included a mini lecture, experiments, and mini discussions. In 2019, we reduced the number of themes to four; we retained four themes from the 2018 course and omitted other three. This reduction enabled us to increase the course time for each theme from one day to two days: each theme lasted two days (approximately sixteen hours); each course contained a mini lecture, experiments, and mini discussions on the first day, and more discussions and report preparations on the second day. The 2019 class included the following topics: conduction through frog sciatic nerve stimulation (hereafter referred to as nerve), skeletal muscle contractions of the frog gastrocnemius (muscle), frog heart electrocardiograms (ECG), and gonadotropin-dependent ovulation of rabbits (reproduction).

### Students

In both 2018 and 2019, ~130 students took this course. They were assigned to undertake a theme in a separated group, and required to understand the physiological significance of each theme by performing the basic physiological experiment and discussion. After completing one theme, they proceeded to take all four themes serially. The instructors comprised the department’s educational staff (professors, associate professors, and assistant professors), who accompanied and provided aid to the students. In 2018 (1d course), ~17 students were assigned to undertake the same theme, except for one theme (urinary theme) where ~ 34 students were exceptionally assigned. The assigned group was further separated into several small groups (~ seven students per small group) where students shared instruments to learn cooperatively. In 2019, ~ 34 students were assigned to take the same theme and took the course over two days. The ratio of students to instructors was made equal in both years: ~17:1 ~2 and ~34:2 ~4 per theme for 2018 and 2019, respectively.

### Questionnaire

**Fig. 1:**
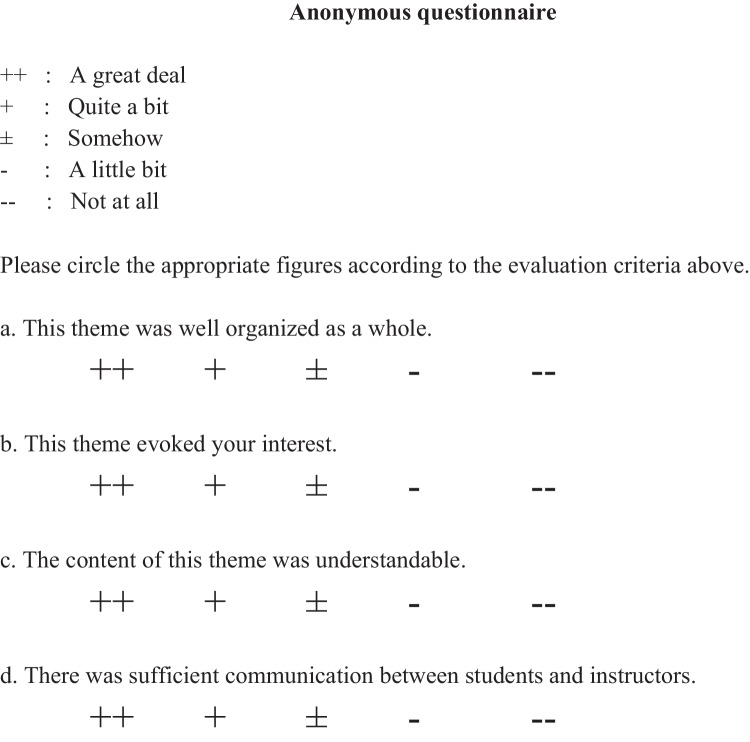
Self-administered questionnaire. The second-year undergraduate medical students voluntarily and anonymously submitted the self-administered questionnaire after completing all the tasks in each theme. Our questionnaire contains questions regarding several issues, including interest in the theme, understanding of the theme, communication with instructors, and general evaluations of the theme

Our questionnaire involved several topics for assessment, including interest in the theme, understanding of the theme, communication with instructors, and general evaluation of the theme (Fig. 1). Students voluntarily and anonymously submitted the questionnaire after completing all the tasks in each theme. Most students (89 and 97 students in 2018 and 2019, respectively) responded to our questionnaire. The ratio of the submitted questionnaires varied from approximately 65 to 70%, according to the theme and year, and the difference of the sample size was negligible. Every question was evaluated on a 5-point Likert scale and subsequently analyzed.

### Statistical Analysis

We averaged the points on the Likert scales and used unpaired Student’s *t*-test to assess statistically significant differences.

### Ethics

We obtained the research ethics approval from the Ethical Review Board of Saitama Medical University (No. 911). All animal experiments in the physiological class were approved and in accordance with the animal care committee at Saitama Medical University (No. 2661, 2662, 2663).

## Results

### Evaluations of Every Theme on a 5-Point Likert Scale

**Fig. 2:**
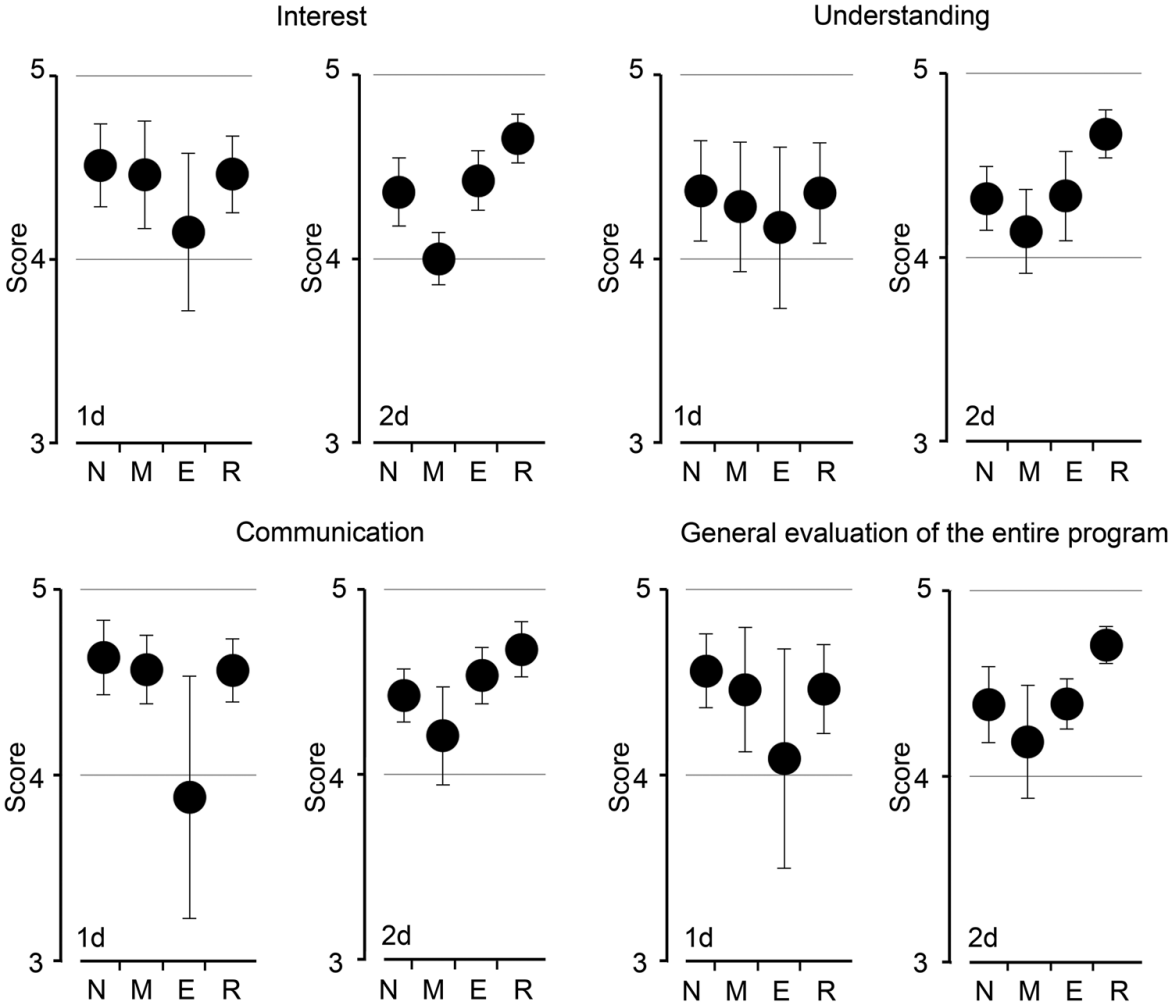
Evaluation of the self-administered questionnaire on a 5-point Likert scale. Students assessed the physiological practice course in terms of interest, understanding, communication, and general evaluation, using a 5-point graded Likert scale after completing each theme: nerve (**N**), muscle (**M**), ECG (**E**), and reproduction (**R**). The scores for the two years (1d and 2d courses in 2018 and 2019, respectively) were averaged among the identical themes and year

The learning “schedule” during each theme differed between 2018 and 2019, whereas learning “content” of the theme was essentially the same (i.e., the difference between the two years is the course time spent for each theme). Of ~ 180 students (89 and 97 students in 2018 and 2019 year, respectively), most of the students voluntarily responded to our questionnaire. The percentage of the responding students ranged from ~ 65 to ~ 70%, according to the theme and year, and the difference of the sample size was negligible. Overall, the average scores revealed that every question was rated highly (Fig. 2), indicating that the students evaluated the class favorably. For interest in the theme, the average score varied between 4.1–4.5 (1d) and 4.3–4.7 (2d), indicating that the educational content successfully evoked significant interest. For understanding, the scores varied between 4.1–4.4 (1d) and 4.2–4.7 (2d), confirming that the content was understandable. The average scores for the communication question varied between 3.9–4.6 (1d) and 4.2–4.6 (2d), indicating that students communicated well with their instructors. Finally, the average general evaluation scores varied between 4.1–4.6 (1d) and 4.2–4.7 (2d), suggesting that the program was, as a whole, favorably assessed. We initially considered that some scores, particularly for understanding and communication, increased in the extended course time situation (2d). However, the average scores were overall similar between the two years, and therefore, we subsequently analyzed the score in more detail (see below “Changes in Score-Distribution Patterns of the Likert Score by Extending Course Time” section).

### Decrease in the 2d Course’s Standard Deviation Values

**Fig. 3:**
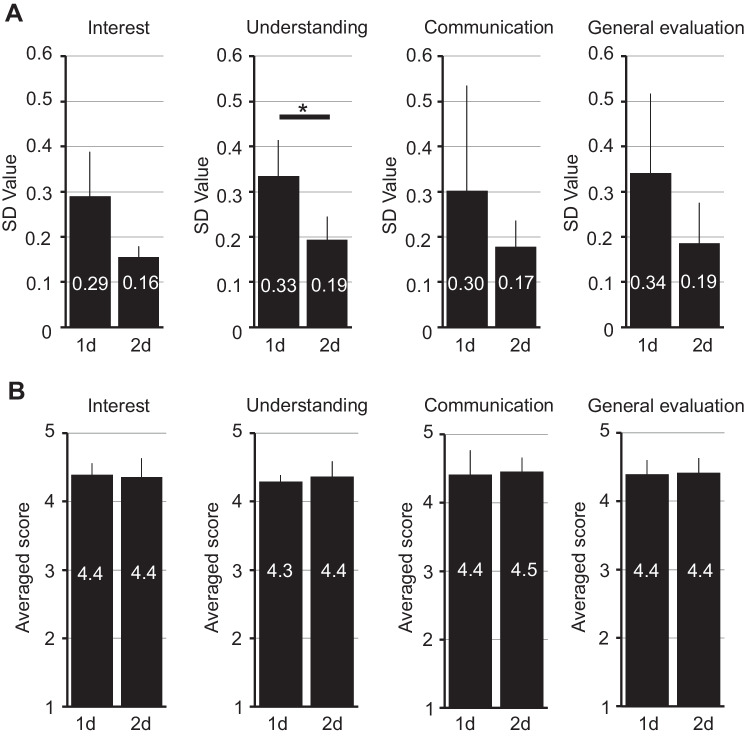
Average Likert score comparisons between 1 and 2d. **A** The standard deviation values (i.e., as pure numbers) of each theme’s Likert scores were averaged among the corresponding year, and analyzed between the 1d and 2d courses (i.e., 1d for 2018 and 2d for 2019) (mean ± SD, *n* = 4; *: *p* = 0.026, Student’s *t*-test, *n* = 4). The average scores of the values are highlighted in the bar of each graph. **B** The four themes’ Likert scores were averaged among the corresponding year, and analyzed between the 1d and 2d courses. The average scores are highlighted in the bar of each graph

Instead of the average scores, the standard deviation (SD) values of every score apparently changed in the 2d course (Fig. 2). For interest, the average SD value decreased from 0.21–0.29 (1d) to 0.13–0.19 (2d). For understanding, it decreased from 0.27–0.44 (1d) to 0.13–0.24 (2d), and for communication, it decreased from 0.18–0.65 (1d) to 0.14–0.26 (2d). For the general evaluation, the SD value decreased from 0.20–0.59 (1d) to 0.10–0.30 (2d) (Fig. 2, Supplementary Table S1). To analyze further, we averaged the SD values for each year and compared the 1d (2018) and 2d (2019) courses (Fig. 3A). For every question, the averaged SD values decreased in the 2d course situation: (i) interest: 0.29 (1d) to 0.16 (2d), (ii) understanding: 0.33 to 0.19, (iii) communication: 0.30 to 0.17, and (iv) general evaluation: 0.34 to 0.19. This result demonstrates that the SD values changed dramatically through the extended course time. This substantial decrease in the SD value led us to consider that the extension of the course from 1 to 2d promoted the dissemination of educational contents to students more efficiently. Thus, we considered the average questionnaire scores to be different between the 1d and 2d courses. However, when we reanalyzed the average scores for each question, we found that the change was not significant: (i) interest: 4.4 (1d) to 4.4 (2d), (ii) understanding: 4.3 to 4.4, (iii) communication: 4.4 to 4.5, and (iv) general evaluation: 4.4 to 4.4 (Fig. 3B).

### Changes in Score-Distribution Patterns of the Likert Score by Extending Course Time

**Fig. 4:**
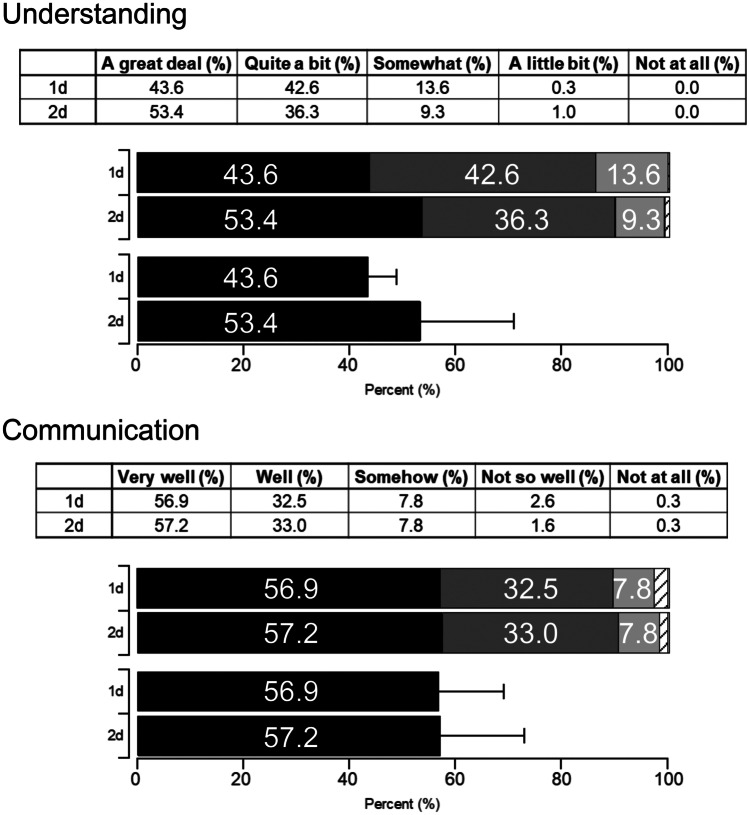
Distribution pattern comparisons of each Likert score between 1 and 2d. (Upper) The ratios of every Likert scores for two questions (understanding and communication) were compared between the 1d and 2d courses. The ratios are highlighted in the bar of each graph. (Lower) The highest points of each question increased in 2d: 43.6% for 1d and 53.4% for 2d in the understanding question (mean ± SD, *p* = 0.32. Student’s *t*-test, *n* = 4)

Figure 3B shows that every question’s average scores were not significantly different between the 1d and 2d courses. We considered that this lack of change may stem from the fact that the original scores were comparatively high. Thus, we reanalyzed the questionnaire in greater detail and compared the ratios of each Likert point between the 1d and 2d courses (Fig. 4), particularly for understanding and communication, as these two factors mostly reflected the educational content’s infiltration. The highest points of each question increased in the 2d course: from 43.6% (1d) to 53.4% (2d) for understanding and 56.9% (1d) to 57.2% (2d) for communication. Accordingly, the course time extension indeed increased the ratio of students that had the highest Likert points. However, the difference in the ratios for understanding was not statistically significant, possibly due to the small number of compared themes (*n* = 4, *p* = 0.32, Student’s *t*-test). In addition, another explanation may be that there were fewer low scores in the 2d course than the 1d course, even though this change in the population did not cause a significant increase in the averaged score. Consequently, this result revealed that the course time extension promoted slightly higher Likert scores for understanding but not for communication.

## Discussions

### Facilitatory Effect of Extending the Course Duration on Educational Content Dissemination

**Fig. 5:**
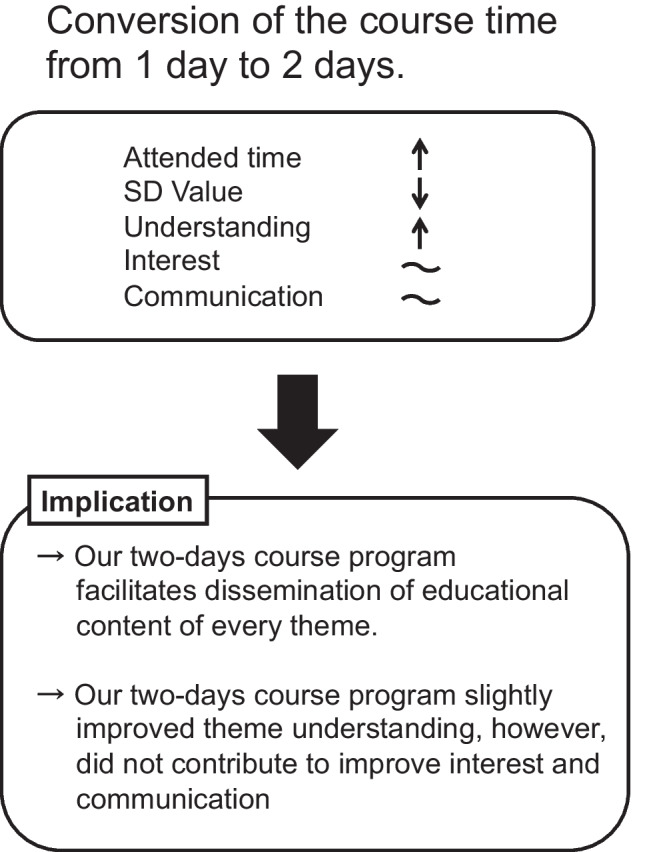
Study summary. In this study, we extended the course duration from one to two days. In the 2d course, students were present in class for a longer time (attended time was increased). Our analyses indicated a profound decrease in the SD values for every question in the self-administered questionnaire and the average score for understanding slightly increased. However, the average scores for interest and communication were similar in both course periods. These results implied that extending the course duration helps to convey educational contents more efficiently to every student; however, this extension did not, at least with the present resources, affect the learning outcomes. We considered that instead of extending the course length, we may need to develop more attractive educational materials, more experimental instruments, and/or better instructor skills to evoke more positive outcomes for the students

In general, the score of the self-administered questionnaire varied according to the individuals. This may depend on the way in which the educational content reaches the student (herein referred to “dissemination” of educational content). In turn, this dissemination is influenced by the student’s motivation or attitudes toward the class, and their circumstances and feelings at the time of the class. Therefore, the questionnaire scores tend to be distributed in a dispersed manner, which was confirmed by the greater SD values in the 1d course (Fig. 2). On the other hand, the SD values of every question in the 2d course significantly decreased (Fig. 3A). This change in SD values indicated that the questionnaire scores were distributed in a more compact manner, suggesting that the educational content was imparted more efficiently to students in the 2d course, regardless of how they grasped the content (favorably or unfavorably). To our knowledge, as there are no reports directly investigating whether the course time length correlates with similar questionnaire scores, our results should be carefully considered. However, we consider that the 2d course, at least in part, helps to facilitate dissemination of the educational content to students (see Fig. 5).

### Course Time’s Facilitatory Effect on Understanding the Educational Content

Previous studies have suggested that the interactive educational approach promotes a more effective understanding of the course content and improves physiology knowledge retention capacity [6, 15]. Therefore, extension of the course duration may contribute to better understanding for students. In addition, we considered that this change decreases the burden of cognitive load that affects learning outcomes [16]. By extending the course duration from 1 to 2d, the speed of learning will slow down; therefore, the burden on working memory is reduced, which may contribute to learn and consolidate the physiological knowledge. Overall, the change implemented in this study would be beneficial for students at the expense of reducing the number of themes. Based on these considerations, we initially considered that our interactive 2d program would improve the understanding scores in our questionnaire. The results revealed that the ratio of students who had high scores did increase in the 2d course. This suggests that 2d program improved theme understandings slightly (Fig. 5), perhaps due to the increased interactive discussion time on the second day. In other words, the 2d course allocated a substantial amount of time to long and interactive discussions between students and instructors, which contributed to students’ understanding. However, the result was only a slight change and the difference was not significant in the overall average score, which also implied that longer and more interactive strategies should be attempted to obtain substantially better student outcomes.

### Course Time’s Effects on Learning Interest Outcome

The interest scores in the questionnaire remained unchanged between the two years, suggesting that course time extension may not be a strongly effective factor for raising students’ interest. Rather, centering on their motivations for learning is the key to increasing interest, which would lead to better learning activities and outcomes [17]. A student can be intrinsically and/or extrinsically motivated. Intrinsic motivation generally means that the educational content is interesting to the student. This depends on how it appeals to their imagination and already existing knowledge. Extrinsic motivation, on the other hand, depends on what and how instructors convey information with their words and demonstrations. In this study’s courses, the educational contents were essentially the same across the two years, suggesting that the course potentially evoked similar levels of the intrinsic motivation. Further, the corresponding instructors for each theme remained the same, suggesting similar levels of extrinsic motivation. Thus, instead of changing course time lengths, we may need to create more attractive educational content and/or further develop instructors’ skills to evoke more student interest.

### Communication and General Evaluation

We initially expected to observe an increase in the communication scores of the 2d course, as students participated in the course for a longer period and had longer discussions with the instructors. However, there was no significant difference in the overall scores (4.4 and 4.5 for 1d and 2d, respectively; Fig. 3B) and the high-score ratios (56.9% and 57.2% for 1d and 2d, respectively; Fig. 4). In addition, the overall score for general evaluation of the theme also showed no difference between the 1d and 2d courses. This implies that increased attendance time does not significantly contribute to communication between students and instructors and general evaluations (Fig. 5). This result may be because that the number of students per accompanied instructor was the same for both courses (approximately eight students per instructor in both programs). Increasing the number of instructors for students may be a more efficient way to raise communication levels and produce better general evaluations.

## Conclusion

In this study, we aimed to identify an effective way to maximize undergraduate medical students’ learning outcomes in the physiological practice course. We examined whether the increased course length leads to improvements in these learning outcomes using self-administered questionnaires. Unfortunately, we could not find strong differences in these scores of the questionnaire between the 1d and 2d courses. However, there was a slight increase in the evaluation of understanding and a substantial decrease in the SD values of every question in the 2d program. Thus, we concluded that extending the course length helped to facilitate dissemination of educational content for every theme.

## Supplementary Information

Below is the link to the electronic supplementary material.

Supplementary file1 (PDF 126 KB)

## Data Availability

All data needed to evaluate the conclusions in the paper are present in the paper.
