# Supplementary material for: Facilitatory Effect of Extending the Course Duration on Dissemination of Educational Content
Source: Med Sci Educ. 2022 May 7;32(3):641–8. doi: 10.1007/s40670-022-01563-4 (PMC9270530; doi:10.1007/s40670-022-01563-4)
Supplement: Supplementary file 1 — Supplementary file1 (PDF 126 KB) [file 40670_2022_1563_MOESM1_ESM.pdf]

Supplementary Material for Satoh et al.

Supplementary Table S1

Interest

Theme	Course	Average	S.D.	*p*-value
Nerve	1d	4.5	0.23	0.25
	2d	4.4	0.19	
Musle	1d	4.5	0.29	0.13
	2d	4	0.14	
ECG	1d	4.1	0.42	0.14
	2d	4.4	0.16	
Reproduction	1d	4.5	0.21	0.1
	2d	4.7	0.13	

Understanding

Theme	Course	Average	S.D.	*p*-value
Nerve	1d	4.4	0.27	0.7
	2d	4.3	0.17	
Musle	1d	4.3	0.35	0.42
	2d	4.1	0.23	
ECG	1d	4.2	0.43	0.42
	2d	4.3	0.24	
Reproduction	1d	4.4	0.27	0.03
	2d	4.7	0.1	

Communication

Theme	Course	Average	S.D.	*p*-value
Nerve	1d	4.6	0.19	0.07
	2d	4.4	0.14	
Musle	1d	4.6	0.18	0.06
	2d	4.2	0.26	
ECG	1d	3.9	0.65	0.02
	2d	4.5	0.15	
Reproduction	1d	4.6	0.17	0.29
	2d	4.7	0.15	

General evaluation

Theme	Course	Average	S.D.	*p*-value
Nerve	1d	4.6	0.2	0.2
	2d	4.4	0.21	
Musle	1d	4.5	0.3	0.2
	2d	4.2	0.3	
ECG	1d	4.1	0.6	0.2
	2d	4.4	0.13	
Reproduction	1d	4.5	0.2	0.0
	2d	4.7	0.1	

Supplementary Table S1 Analyzed data of the self-administered questionnaire
Analyzed data of Likert score (mean, S.D., *p*-value of unpaired Student’s *t*-test) are shown.
